# Nucleic Acid Extraction from Synthetic Mars Analog Soils for *in situ* Life Detection

**DOI:** 10.1089/ast.2016.1535

**Published:** 2017-08-01

**Authors:** Angel Mojarro, Gary Ruvkun, Maria T. Zuber, Christopher E. Carr

**Affiliations:** ^1^ Department of Earth, Atmospheric and Planetary Sciences, Massachusetts Institute of Technology, Cambridge, Massachusetts.; ^2^ Department of Molecular Biology, Massachusetts General Hospital, Boston, Massachusetts.; ^3^ Department of Genetics, Harvard Medical School, Boston, Massachusetts.

## Abstract

Biological informational polymers such as nucleic acids have the potential to provide unambiguous evidence of life beyond Earth. To this end, we are developing an automated *in situ* life-detection instrument that integrates nucleic acid extraction and nanopore sequencing: the Search for Extra-Terrestrial Genomes (SETG) instrument. Our goal is to isolate and determine the sequence of nucleic acids from extant or preserved life on Mars, if, for example, there is common ancestry to life on Mars and Earth. As is true of metagenomic analysis of terrestrial environmental samples, the SETG instrument must isolate nucleic acids from crude samples and then determine the DNA sequence of the unknown nucleic acids. Our initial DNA extraction experiments resulted in low to undetectable amounts of DNA due to soil chemistry–dependent soil-DNA interactions, namely adsorption to mineral surfaces, binding to divalent/trivalent cations, destruction by iron redox cycling, and acidic conditions. Subsequently, we developed soil-specific extraction protocols that increase DNA yields through a combination of desalting, utilization of competitive binders, and promotion of anaerobic conditions. Our results suggest that a combination of desalting and utilizing competitive binders may establish a “universal” nucleic acid extraction protocol suitable for analyzing samples from diverse soils on Mars. Key Words: Life-detection instruments—Nucleic acids—Mars—Panspermia. Astrobiology 17, 747–760.

## 1. Introduction

Widespread synthesis of the known building blocks of life, that is, amino acids and nucleobases, within the early planetary nebula (Nuevo *et al.,* 2009, 2012; Ciesla and Sandford, 2012) may have biased life as we know it toward the utilization of nucleic acids as the basic medium for information storage and transference (*e.g.,* deoxyribonucleic acid—DNA—and ribonucleic acid—RNA). Recent work also demonstrates synthesis of ribose and related sugars under conditions similar to those of the late solar nebula (Meinert *et al.,* 2016). Life on Mars, if it developed independently, may have adopted a similar medium of information storage and readout transfer (DNA, RNA), although this assumes that there is a unitary chemical solution to the problem of genetic transmission. We propose that, if life has transferred between Earth and Mars, perhaps billions of years ago, the highly evolved tools of modern genomics and metagenomics could be applied to the search for extant or preserved life on Mars (Ruvkun *et al.,* 2002, Isenbarger *et al.,* 2008).

Common ancestry of life on Mars with life on Earth could have been mediated by the estimated billion tons of rock transferred between Mars and Earth during the Late Heavy Bombardment period (Gladman and Burns, 1996; Gladman *et al.,* 1996). In this scenario, viable microbes may have been transported between planets within meteoroids, some of which experienced nonsterilizing transfers in space (Weiss *et al.,* 2000; Fritz *et al.,* 2005; Shuster and Weiss, 2005) and tolerable shock pressures and heating upon planetary entry (Ruvkun *et al.,* 2002, Horneck *et al.,* 2008, Isenbarger *et al.,* 2008). In such a case, comparing genetic sequencing data from martian “life” with conserved (Makarova *et al.,* 1999; Harris *et al.,* 2003) genetic sequences from known Earth life (*i.e.,* the ribosomal RNA) (Woese *et al.,* 1975) could discriminate forward contamination from true life detection and establish a possible common ancestry (Ruvkun *et al.,* 2002; Isenbarger *et al.,* 2008).

**FIG. 1.**
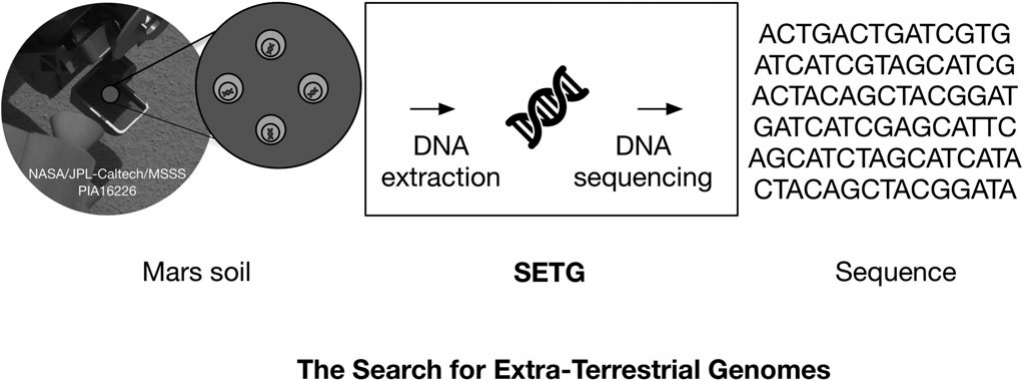
Overview of the SETG instrument. Soils collected by a sample-handling system will be processed; any extracted nucleic acids will then be conditioned and sequenced *in situ*. Any resulting sequences can be compared to all known Earth sequences to determine if such sequences represent forward contamination or putative martian life. Image adapted from PIA16226 (NASA/JPL-Caltech/MSSS).

We are developing an automated nucleic acid extraction and sequencing instrument (Lui *et al.,* 2011; Carr *et al.,* 2013a, 2013b, 2016), Search for Extra-Terrestrial Genomes (SETG), that is capable of analyzing a variety of environmental samples relevant to the search for evidence of life (related or otherwise to life on Earth) on Mars as well as on other habitable bodies in the Solar System (Fig. 1). On Earth, metagenomic sequencing of DNA extracted from environmental samples has been an invaluable tool for identifying new and unique “unculturable” microorganisms (Schloss and Handelsman, 2005). However, complex soil matrices like those containing iron oxides, for example, have strong inhibitory side effects toward nucleic acid extraction due to problems of competitive binding (Jiang *et al.,* 2012; Hurt *et al.,* 2014) and the production of destructive hydroxyl radicals from Fe^3+^ and Fe^2+^ redox cycling (Imlay and Linn, 1988; Imlay *et al.,* 1988). Additional inhibition may emerge in the presence of silicates (Volossiouk *et al.,* 1995; Melzak *et al.,* 1996; Trevors, 1996), clay minerals (Greaves and Wilson, 1969), high concentrations of salt minerals, and acidic conditions (Henneberger *et al.,* 2006). These soil-DNA interactions are especially problematic in low-biomass terrestrial environments (Barton *et al.,* 2006; Azua-Bustos *et al.,* 2012, 2015) and pose similar challenges for the extraction of nucleic acids from Mars analog soils (Navarro-Gonzalez *et al.,* 2003; Mojarro *et al.,* 2016).

Extensive literature exists on how to mitigate soil-DNA interactions and increase DNA extraction yields from a variety of soil species (*e.g.,* Purdy *et al.,* 1996; Takada-Hoshino and Matsumoto, 2004; Barton *et al.,* 2006; Henneberger *et al.,* 2006; Herrera and Cockell, 2007; Direito *et al.,* 2012; Lever *et al.,* 2015). The use of competitive binders (skim milk, RNA, poly-dIdC) (Volossiouk *et al.,* 1995; Takada-Hoshino and Matsumoto, 2004; Barton *et al.,* 2006), sample dialysis (Barton *et al.,* 2006), and chelation of calcium and iron inhibitors through the use of ethylenediaminetetraacetic acid (EDTA) or ethylene glycol tetraacetic acid (EGTA) (Wade and Garcia-Pichel, 2003; Barton *et al.,* 2006) in combination with traditional soil nucleic acid extraction kits (Yakimov *et al.,* 2002; Wade and Garcia-Pichel, 2003; Takada-Hoshino and Matsumoto, 2004; Walker *et al.,* 2005; Barton *et al.,* 2006; Brown and Wolfe, 2006; Henneberger *et al.,* 2006) is an effective method of extracting PCR-quality DNA from various complex terrestrial environments. These solutions, however, require involved steps, additional equipment, and large volumes of reagents that would increase the complexity and size of an *in situ* SETG instrument on Mars.

Our goal for SETG is to generate a versatile nucleic acid extraction protocol capable of processing samples from diverse environments on Mars (Fig. 1). A typical benchtop DNA extraction protocol requires bead beating, centrifugation, and frequent spin-column nucleic acid purification steps, while archaic protocols require freezing samples in liquid nitrogen, manual grinding with a mortar and pestle, phenol/chloroform nucleic acid isolation, and several alcohol purification steps. Although the aforementioned extraction methods have been proven effective, we wish to develop a system that employs minimal extraction steps and reagent volumes, requires low power, and is compact for variable spaceflight applications involving rover/lander or human operations. In this study, we utilize PureLyse Bacterial gDNA Kits (ClaremontBio, 01.351.06), which are miniature (dime-sized), low-power (6 V), rapid (3 min protocol) solid-phase nucleic acid extraction devices, as the precursor to a “mature” SETG extraction module. Additionally, PureLyse-related hardware has been validated in orbit as part of NASA's WetLab-2 platform aboard the International Space Station to extract and purify RNA from cells (Robert Doebler, personal communication, 2016).

**FIG. 2.**
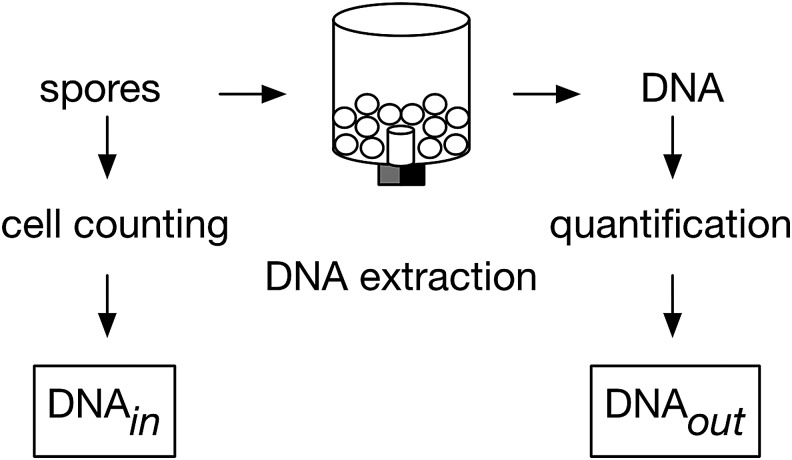
Simplified experimental overview of extraction of spore DNA. Counted DNase-treated spores of *Bacillus subtilis* ATCC 6633 (DNA_in_) are loaded into PureLyse, processed, and yield DNA quantified by a dsDNA-specific fluorometric assay (DNA_out_).

To characterize PureLyse for SETG's application, we challenge DNA extraction performance with spores of *Bacillus subtilis* ATCC 6633 (Fig. 2), a hardy microorganism with spaceflight heritage known to survive exposure in orbit (Horneck *et al.,* 2012) and in synthetic Mars analog soils under martian temperature, pressure, and radiation (Schuerger *et al.,* 2012). We first test the recovery of *Escherichia coli* DNA and the extraction of *B. subtilis* spore DNA from synthetic Mars analog soils in order to characterize soil-DNA interactions that may adversely affect DNA extraction performance on Mars. We then develop modified DNA extraction protocols to mitigate the identified soil-DNA interactions. Modified extraction protocols were based on 50 mg soil samples, the upward sample loading capacity of PureLyse. Finally, we determine the purity of extracted DNA via quantitative real-time polymerase chain reaction (qPCR) as a proxy for sequencing, which would be used on the full SETG instrument, to verify that no compounds were coextracted from the soil that would inhibit DNA sequencing.

## 2. Materials and Methods

### 2.1. Approach

Here, we show that a PureLyse-like system may be adapted to extract nucleic acids from martian or Mars-like soils. Our two primary experiments are focused on “recovery of free DNA” and “extraction of spore DNA” from synthetic Mars analog soils. The purpose of the “recovery of free DNA” experiment is to establish interactions that may occur between free DNA, defined as extracellular DNA able to freely interact in a solvent, and soil. In contrast, the purpose of the “extraction of spore DNA” experiments is to determine PureLyse lysis and extraction performance of DNA insulated by an endospore coat (*i.e., B. subtilis* spores) in soils. These experiments culminate in the generation of extraction modifications that mitigate soil-DNA interactions and maximize DNA yields from spore DNA in soil. We consider a DNA extraction yield of 5% or greater to be adequate for subsequent sequencing; this would allow us to achieve, at a minimum, our sensitivity target of 1 ppb, which corresponds to ∼10^4^ *B. subtilis* spores in a 50 mg sample (Carr *et al.,* 2016) as we approach a mature SETG instrument. Here, we focus on higher cell counts and leave it to future work to demonstrate that a similar extraction yield can be achieved at lower cell counts.

Finally, the purpose of the “soil carryover” experiment is to evaluate the purity of extracted DNA for the presence of soil contaminants or “carryover” that may inhibit downstream sequencing; as a proxy, we utilized qPCR, which relies on polymerase and serves as a cost-effective proxy for current nanopore sequencing technologies, which use polymerase (Fuller *et al.,* 2016) or helicase (Derrington *et al.,* 2016) enzymes. Of note, qPCR is the standard method used to evaluate the quality of DNA molecules prior to traditional next-generation (*e.g.,* Illumina, Ion Torrent) sequencing.

### 2.2. Synthetic Mars analog soils

Six synthetic Mars analog soils and one lunar analog soil, representing unaltered martian basalt, were used. Five of the synthetic Mars analog soils were prepared in accordance with *in situ* measurements (*e.g.,* X-ray diffraction) from rover and lander surface sites as described by Schuerger *et al.* (2012). The five synthetic Mars analogues represent the salt-rich soils of Gusev Crater (Ming *et al.,* 2006; Morris *et al.,* 2008), jarosite-containing acidic soils of Meridiani Planum (Klingelhöfer *et al.,* 2004; Morris *et al.,* 2006), carbonate-rich alkaline soils of Chryse Planitia (Clark *et al.,* 1982; Wänke *et al.,* 2001), weakly alkaline and perchlorate-rich soils of Vastitas Borealis (Ming *et al.,* 2008), and the pervasive global aeolian soils (Bell *et al.,* 2000). These synthetic Mars analog soils are henceforth referred to as salt, acid, alkaline, perchlorate, and aeolian, respectively. In addition, a commercially available spectral analogue of Mars (Orbitec, JSC Mars-1A) (Allen *et al.,* 2000) and lunar analogue (Orbitec, JSC-1A) (McKay *et al.,* 1994), designated JSC and basalt, were also used.

**FIG. 3.**
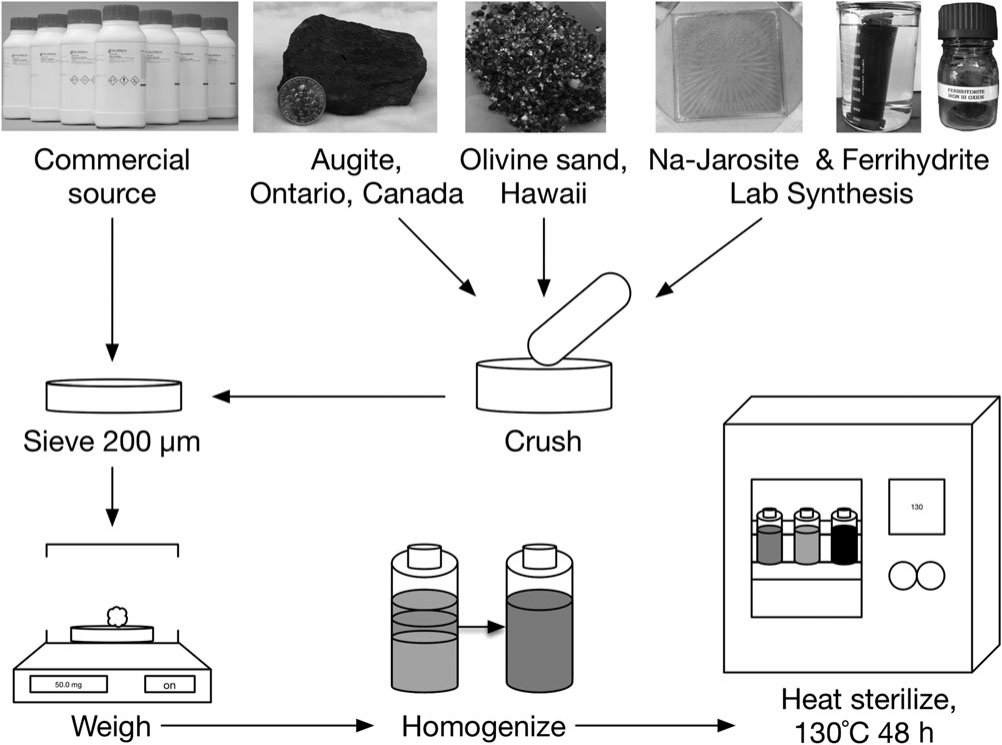
Overview of synthetic Mars analog synthesis process, adapted from Schuerger *et al.* (2012). Synthetic samples are controlled, reproducible, and simulate various martian environments.

All minerals and salts were commercially sourced from Sigma-Aldrich (calcium carbonate—481807, calcium phosphate—04231, ferric sulfate—307718, gypsum—237132, halite—S9888, hematite—310050, kieserite—434183, magnesium chloride—208337, Ti-magnetite—310069, magnesite—63062, sodium carbonate—451614, sodium perchlorate—310514, sodium sulfate—204447) with the exception of pyroxene, olivine, anhydrite, ferrihydrite, and natrojarosite (Fig. 3). Pyroxene was purchased from Ward's Science (Catalog #466474). Olivine sand was collected from Papalōlea beach, near South Point, Hawaii, in accordance with the work of Schuerger *et al.* (2012) and cleaned with a 0.2 *M* sodium acetate solution (Doug Ming, personal communication, 2015). Anhydrite was produced by dehydrating gypsum (Sigma-Aldrich, 237132) at 300°C overnight (Atoji, 1959), and 2-line ferrihydrite was chemically synthesized from reagent-grade iron (III) nitrate (Sigma-Aldrich, 216828) (Schwertmann and Cornell, 2008). Lastly, natrojarosite was synthesized from reagent-grade iron (III) sulfate (Sigma-Aldrich, 307718) and sodium sulfate (Sigma-Aldrich, S9627) (Drouet and Navrotsky, 2003).

Individual minerals, salts, and commercial analog soils were hand-crushed with a Coorstek alumina mortar and pestle and passed through a 200 μm stainless steel sieve (Fig. 3) (Schuerger *et al.,* 2012). Mineral and salt powders were then individually weighed and then mixed and homogenized in 100 g batches to represent the acid, aeolian, alkaline, perchlorate, and salt synthetic analog soils (Schuerger *et al.,* 2012) in addition to the JSC and basalt soils (Fig. 3). All soils were heat-sterilized at 130°C for 48 h prior to any experimentation in a Fisher Science Isotemp 282A vacuum oven (Fig. 3), cooled overnight, and stored in air-tight borosilicate bottles (Corning, 1395-100) at room temperature (Schuerger *et al.,* 2012).

### 2.3. *Bacillus subtilis* spores

Spore suspensions of *Bacillus subtilis* (ATCC 6633) were purchased from NAMSA (Part# SBS-08), and spore titer was verified by plating on lysogeny broth agar media followed by counting colonies after incubation at 37°C for 24 h. Spores were treated with DNAse I (New England Biolabs, M0303L) prior to any extractions.

### 2.4. DNA yield quantitation

We define DNA yield from our experiments as

\begin{align*}
 { \rm { DN } } { { \rm { A } } _ { { \rm { yield } } } } \% = 100 { \frac { { \rm { DN } } { { \rm { A } } _ { { \rm { out } } } } }  { { \rm { DN } } { { \rm { A } } _ { { \rm { in } } } } } } 
\end{align*}

We assume a single genome copy (3,987,576 base pairs) per DNAse I-treated spore of *B. subtilis* for calculating DNA_in_. DNA_out_ was quantified using a double-stranded, DNA-specific fluorometric assay (Invitrogen, Qubit dsDNA HS Assay Kit, Q32854) and a Qubit 2.0 fluorometer (Invitrogen, Q32866, limit of detection of 0.1 ng/mL, dsDNA).

### 2.5. PureLyse

The PureLyse Bacterial gDNA Extraction Kit is a Claremont Biosolutions OmniLyse mechanical cell disruption module (30,000 rpm bead beating), coupled with proprietary binding and elution buffers, that enables rapid solid-phase extraction of nucleic acids (including RNA). The standard off-the-shelf protocol advises 2 min lysis with 1× binding buffer solution (100 μL 8× binding buffer and 700 μL water), 45 s wash with 3 mL of 0.25× binding buffer wash (∼100 μL 8× binding buffer and 2.9 mL water), and a 1 min elution with 200 μL 1× elution buffer.

### 2.6. Recovery of free DNA from synthetic Mars analog soils

**FIG. 4.**
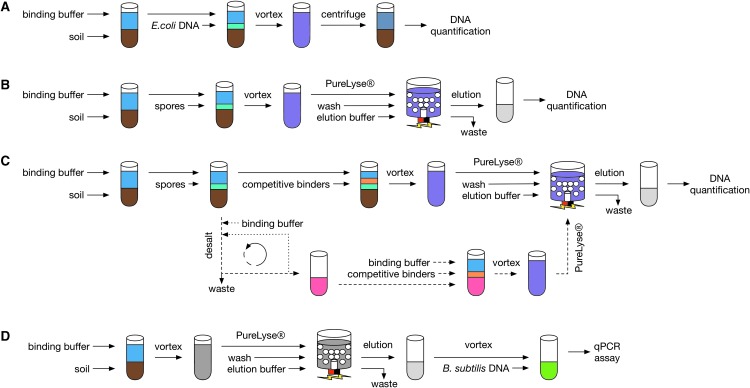
Experiment overview. (**A**) Recovery of free DNA from synthetic Mars analog soils. (**B**) Baseline extractions of spore DNA from synthetic Mars analog soils using the standard PureLyse protocol. (**C**) Modified extractions of spore DNA from synthetic Mars analog soils. (**D**) Soil carryover qPCR assay.

Purified *Escherichia coli* DNA (USB Corporation, #14380, 1859 ng) was added to each set of microcentrifuge tubes containing 0–50 mg (0, 10, 20, 30, 40, 50 mg, *n* = 3) of synthetic Mars analog soil and 800 μL of 1× PureLyse binding buffer solution. The microcentrifuge tube mixtures were homogenized on a digital vortex mixer (VWR, #14005-824) at 1000 rpm for 1 min and immediately centrifuged (Eppendorf, Centrifuge 5418) for 5 min at 10,000 rpm. The amount of recoverable DNA from the supernatant was then measured with a fluorometric assay (Fig. 4A).

### 2.7. Baseline extractions of spore DNA from synthetic Mars analog soils

An estimated 2.2 × 10^8^ spores (about 1 μg of DNA) of *B. subtilis* were processed with 0–50 mg (water control and 10, 20, 30, 40, 50 mg, *n* = 3) of synthetic Mars analog soil following the standard DNA extraction protocol (Fig. 4B). DNA yields were calculated from two combined elutions (200 μL each) with a fluorometric assay.

### 2.8. Modified extractions of spore DNA from synthetic Mars analog soils

As above, equal amounts of *B. subtilis* spores (2.2 × 10^8^, ∼1 μg DNA) were combined with 50 mg of each synthetic Mars analog soil as well as a water control (*n* = 3) and processed with modified DNA extraction protocols developed per analog soil.

Water (control) samples were processed with 40 μL of phenol solution (Sigma, P4557) added to 760 μL of binding buffer solution (100 μL 8× binding buffer and 660 μL water) to create a final 1× binding buffer concentration and 5% phenol solution.

**FIG. 5.**
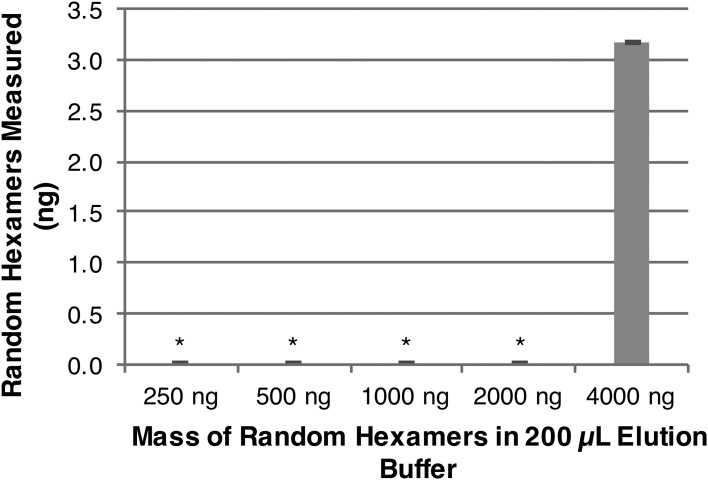
Random hexamer primer detection by a dsDNA-specific fluorometric assay. Random hexamers (250, 500, 1000, 2000, 4000 ng) were added to 200 μL of the PureLyse elution buffer, homogenized, then quantified on a Qubit 2.0 fluorometer (standard error shown, *n* = 3). Random hexamer loads were not detectable until 20 ng/μL (4000 ng in 200 μL), likely due to increased hybridization. These results suggest that using random hexamer primers (less than 20 ng/μL) as competitive binders will not bias DNA yield results with this dsDNA-specific fluorometric assay. *At or below 0.01 ng limit of detection.

Basalt samples were processed with 150 ng of random hexamer primers (Promega, Random C1181). The soil and primers were homogenized for 1 min at 1000 rpm, then processed following the standard DNA extraction protocol (Fig. 4C). Random hexamer primers used here were removed from the elutions (Mojarro *et al.,* 2016) prior to measuring DNA yield with an extra cleaning step (Zymo, Genomic Clean & Concentrator). Subsequent testing demonstrated that random hexamer primer concentrations up to 20 ng/μL are not measurable using the dsDNA-specific fluorometric assay (Fig. 5).

Alkaline soil samples were processed with a modified DNA extraction protocol containing 4× binding buffer solution (400 μL 8× binding buffer and 400 μL water) and 1× wash solution (Fig. 4B).

JSC soil samples were processed inside an anaerobic chamber (Sigma, Atmosphere Bag, Z530212): after flushing the chamber with nitrogen gas, reagents were degassed for 1 h (Butler *et al.,* 1994) in the presence of oxygen scavengers (Thermo Scientific, AnaeroGen, 2.5L) before processing via the standard DNA extraction protocol (Fig. 4B).

Salt soil samples were suspended in 800 μL of 8× binding buffer solution and desalted on a 100K Amicon Ultra column (Z740183) prior to extraction. Samples were then processed with 4× binding buffer solution and 1× wash solution (1.5 mL 8× binding buffer and 1.5 mL water) (Fig. 4C).

Perchlorate and aeolian soil samples were prepared in accordance with the salt soil protocol. Furthermore, perchlorate soil samples included 300 ng of random hexamer primers, while the aeolian soil samples included 500 ng (Fig. 4C) when processed with the 4× binding buffer solution.

Lastly, acid soil samples were also prepared following the salt protocol, but with the following modifications: samples were resuspended in multiple volumes of 8× binding buffer within the 100K Amicon Ultra column and desalted until the color of the waste became clear. Acid soil samples were then extracted using a 4× binding buffer solution that included 2 mg of skim milk (40 mg of skim milk per 1 g of soil, Takada-Hoshino and Matsumoto, 2004) and 2× wash solution (Fig. 4C).

Table 1 compiles the modified DNA extraction protocols developed in this study.

**Table 1.** Summary of Methods for Extracting Nucleic Acids from Mars Analog Soils (50 mg Soil)

*Synthetic sample*	*Model geographic location on Mars*	*Soil characteristics*	*Proposed DNA interaction(s)*	*Modified DNA extraction*	*Baseline yield (%)*	*Modified yield (%)*	*Notes*
Water	—	—	SASPs inhibit DNA extraction	5% phenol extraction	14.7%	43.0%	—
Basalt	Subsurface	Unaltered basalt, ∼50% SiO_2_	Adsorption to silica	150 ng of random hexamer primer	12.2%	13.7%	JSC-1A lunar regolith
Alkaline	Chryse Planitia	Carbonate-rich (Mg, Ca)	Adsorption to silica Binding to Ca^2+^	4× binding buffer	6.62%	11.2%	—
JSC	Global	Highly oxidized	Hydroxyl radical generation DNA adsorption to silica DNA binding to Fe^3+^	Degas reagents/Anaerobic extraction	3.04%	6.39%	JSC Mars-1A spectral analogue
Salt	Gusev Crater	Salt-rich, highly oxidized	High salts DNA adsorption to silica DNA binding to Ca^2+^ DNA binding to Fe^3+^	Desalt soil suspended in 8× binding buffer in 100 kDa column prior to extraction. Resuspend in 4× binding buffer and 1× wash.	1.57%	6.64%	—
Perchlorate	Vastitas Borealis	Weakly alkaline, perchlorate-rich, highly oxidized	High salts DNA adsorption to silica DNA binding to Ca^2+^ DNA binding to Fe^3+^ Hydroxyl radical generation	Desalt soil suspended in 4× binding buffer in 100 kDa column. Re-suspend in 4× binding buffer, 300 ng random primer hexamer, and 1× wash.	0.58%	6.49%	—
Aeolian	Global	Highly oxidized	High salts DNA adsorption to silica DNA binding to Ca^2+^ DNA binding to Fe^3+,2+^ Hydroxyl radical generation	Desalt soil suspended in 4× binding buffer in 100 kDa column. Resuspend in 4× binding buffer, 500 ng random primer hexamer, and 1× wash.	0.23%	5.03%	—
Acid	Meridiani Planum	Highly oxidized, acidic pH 1.42, contains jarosite	DNA destruction by low pH Hydroxyl radical generation DNA adsorption to silica DNA binding to Ca^2+^ DNA binding to Fe^3+^	Desalt soil in 100 kDa column in multiple 8× binding buffer solutions. Re-suspend in 4× binding solution, 2 mg skim milk, and 2× wash.	0%	6.61%	Samples were desalted in continuous 8× binding buffer volumes until the waste color became clear

### 2.9. Soil carryover

A total of 50 mg of synthetic Mars analog soil was processed following the standard DNA extraction protocol (*n* = 3) without *B. subtilis* spores. *Bacillus subtilis* DNA, purified from vegetative cells, was then added to the “blank” elution to achieve a final concentration of 0.2 ng DNA/μL (Fig. 4D). A total of 5 μL of the simulated extract, containing 1 ng of DNA, was then amplified in a 20 μL (total) reaction volume of PerfeCta SYBR Green FastMix and Integrated DNA Technologies Readymade 16s rRNA primers (Universal). Our qPCR mix and 96-well plate were prepared inside an AirClean 600 PCR Workstation by an Andrew Alliance liquid handling robot. Thermocycling conditions were as follows: (1) 94°C for 3 min, (2) 94°C for 45 s, (3) 50°C for 1 min, (4) 72°C for 90 s, (5) plate read (6) repeat steps 2–5 40 times.

## 3. Results

### 3.1. Recovery of free DNA from synthetic Mars analog soil

**FIG. 6.**
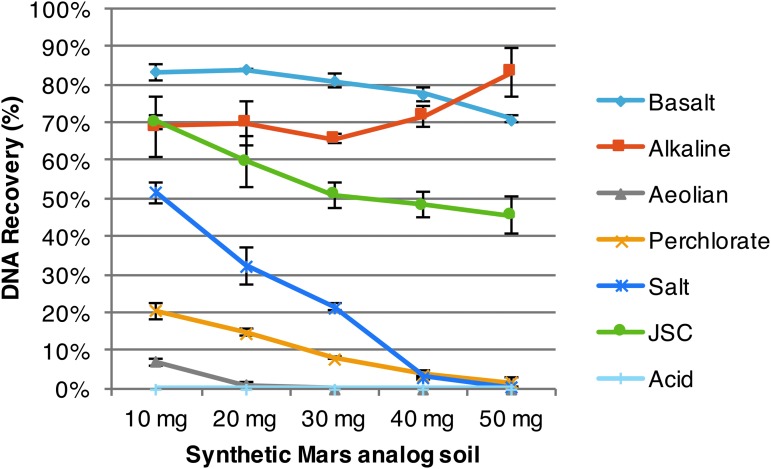
Recovery of free DNA from synthetic Mars analog soils. Purified *Escherichia coli* DNA, ∼2 μg, was added to synthetic Mars analog soils (0–50 mg); DNA recovery profiles identify general soil-DNA interactions, namely competitive adsorption to minerals, binding to free cations, and presumed destruction (standard error shown, *n* = 3).

Approximately 80% of DNA added to the sample was recoverable in the basalt and alkaline, for all quantities of soil tested (Fig. 6). Otherwise, the aeolian, perchlorate, salt, and JSC soils exhibited an inferred linear relationship, where increasing soil mass decreases DNA recovery (Fig. 6). No measurable DNA (lower limit of detection 0.1 ng/mL) was recovered from the acid soils (10–50 mg) (Fig. 6), which may be due to the destruction of DNA through depurination and hydrolysis in low pH (Schroeder *et al.,* 2006; Bernhardt and Tate, 2012).

### 3.2. Baseline and modified extractions of spore DNA from water

**FIG. 7.**
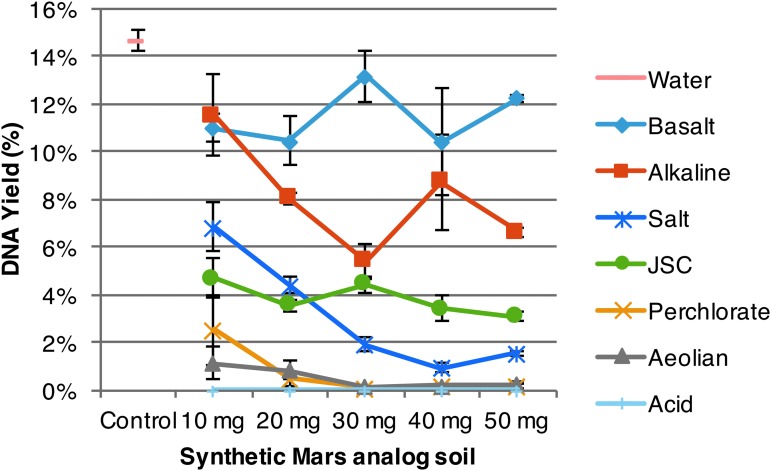
Baseline extraction yields of spore DNA from synthetic Mars analog soils and water. *Bacillus subtilis* spores, 2.23 × 10^8^ (∼1 μg DNA), were added to water (control) and 10–50 mg of basalt, alkaline, aeolian, acid, perchlorate, salt, and JSC (standard error shown, *n* = 3). All soils adversely affect DNA extraction performance; low DNA yield from water is likely due to SASPs that interfere with solid-phase extraction.

**FIG. 8.**
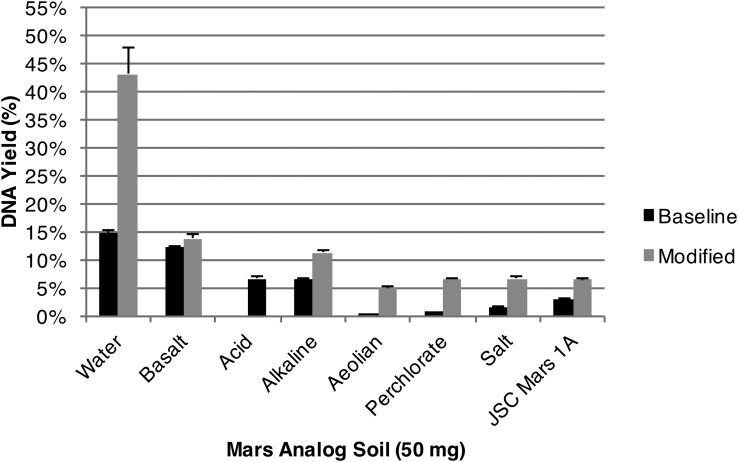
Modified and baseline extraction yields of spore DNA from synthetic Mars analog soils and water. DNA yields from 50 mg of soil (including water control) and 2.23 × 10^8^ *Bacillus subtilis* spores (∼1 μg DNA) were increased with soil-specific modified extraction protocols (standard error shown, *n* = 3). Our results suggest that a combination of desalting and competitive binders may establish a “universal” Mars protocol.

Baseline DNA extractions of *B. subtilis* spores from our water (control) sample produced 14.7% DNA yield (Fig. 7). This result is significantly lower than manufacturer studies of PureLyse that have demonstrated ∼90% DNA extraction yields from *Escherichia coli* (ClaremontBio Solutions, 2011) with multiple elutions. PureLyse kits operate by adsorbing nucleic acids onto zirconium-silicate microbeads via salt bridges that link the phosphate backbone under high salt and moderately acidic (∼3.4 pH) conditions. Our low DNA extraction yields from *B. subtilis* spores may be caused by small acid-soluble proteins (SASPs) that interfere with the extraction process (Setlow and Setlow, 1993; Moeller *et al.,* 2008). If the DNA backbone is in fact bound by these acidic proteins, it will disregard the salt bridges, and DNA will be discarded in the waste. To test this model, “on the fly” protein separation was facilitated with phenol. A 5% final solution (Chomczynski and Sacchi, 1987; Volossiouk *et al.,* 1995) resulted in significantly increased DNA yields from 14.7% to 43% (Fig. 8). We consider the final phenol concentration limiting, as solutions containing greater than 5% phenol dissolved the OmniLyse module.

### 3.3. Baseline extractions of spore DNA from synthetic Mars analog soils

The baseline DNA extractions of *B. subtilis* spores from synthetic analog soils (10–50 mg) showed a similar trend as the free DNA soil recovery assay (Figs. 6 and 7), resulting in decreasing DNA yields with increasing soil mass (Fig. 7). At 50 mg, basalt and alkaline soils moderately affected yields compared to baseline water (control) extractions (Fig. 7) with about 12% DNA yield. Meanwhile, JSC, perchlorate, aeolian, salt, and acid dramatically decreased yields (Fig. 7), less than 3% as observed earlier (Fig. 6).

### 3.4. Modified extractions of spore DNA from synthetic Mars analog soils

Basalt soil sample DNA yields were increased from 12.2% to 13.7% (Fig. 8) to nearly replicate water control yields by employing random hexamer primers as competitive binders to prime DNA adsorption sites prior to cell lysis. Decreased yields in basalt soil samples are likely associated with competitive DNA adsorption to silicates (Melzak *et al.,* 1996; Trevors, 1996; Zhou *et al.,* 1996).

Alkaline soil sample DNA yields were increased from 6.6% to 11.2% (Fig. 8), comparable to unmodified basalt soil DNA yields, by increasing binding buffer concentration. Although nearly identical to basalt, alkaline samples contain 4 wt % (Ca, Mg) carbonate that may bind DNA. Previous studies that focused on carbonate environments (Barton *et al.,* 2006) have utilized EGTA, a calcium-specific chelator, to remove DNA-binding calcium cations (Barton *et al.,* 2006). Although EDTA has a lower calcium affinity (which the binding buffer presumably contains), we increased the binding buffer concentration. At a 4× binding buffer concentration, chelator specificity became negligible.

JSC soil sample DNA yields were increased from 3.0% to 6.4% (Fig. 8). Analysis of the JSC analog soil by Allen *et al.* (2000) revealed a strong Fe^3+^:Fe^2+^ couple, present in a 3:1 ratio, that may lead to the production of destructive hydroxyl radicals (Imlay and Linn, 1988; Imlay *et al.,* 1988) under aqueous conditions. Thereby presumed DNA destruction was prevented by removing dissolved oxygen from our reagents through degassing in an anaerobic chamber.

Salt soil sample DNA yields were increased from 1.6% to 6.6% (Fig. 8) by desalting and without any further treatment with competitive binders. The salt soil is composed of 13.9 wt % salt minerals (calcite, gypsum, kierserite, magnesium chloride) that bind DNA and inhibit elution (occurs under low salt). Successful dialysis techniques utilized by Barton *et al.* (2006) with carbonate-rich samples informed a pre-extraction desalting step.

For aeolian and perchlorate soil samples, the combination of desalting and competitive binders (random hexamer primers) increased DNA extraction yields from 0.2% to 5.0% (Fig. 8) in the aeolian soil and 0.6% to 6.5% in the perchlorate soil samples (Fig. 8). Extraction of DNA from aeolian and perchlorate soil samples when using the unmodified protocol resulted in greatly reduced DNA yields due to presumed production of hydroxyl radicals that destroy DNA (Imlay and Linn, 1988; Imlay *et al.,* 1988) and high concentrations of DNA-binding iron cations (Fe^3+,2+^); (Volossiouk *et al.,* 1995; Takada-Hoshino and Matsumoto, 2004; Barton *et al.,* 2006). Before implementing our optimized protocol, we initially addressed hydroxyl radicals by degassing reagents inside an anaerobic chamber; however, yields minimally increased when compared to JSC soil samples. Comparable yields to an anaerobic treatment resulted when both soils were desalted prior to extraction. We conclude that the majority of DNA is lost due to strong competitive adsorption to iron-bearing minerals (ferrihydrite, hematite, Ti-magnetite) once hydroxyls and free cations have been addressed.

Acid sample DNA yields were increased to 6.6% (Fig. 8) by employing a combination of desalting and skim milk (Takada-Hoshino and Matsumoto, 2004). Initial extractions from the acid soils yielded no detectable DNA due to presumed destruction by high acidity (pH of 1.42, Figs. 7 and 8). Buffering pH prior to extractions resulted in measurable DNA yields, <0.7%, similar to the untreated aeolian or perchlorate soils (Figs. 7 and 8). Desalting mildly improved yields, though high iron concentrations from 27 wt % ferric sulfate, 1 wt % jarosite, and 2.5 wt % hematite resulted in lower extraction yields in comparison to aeolian or perchlorate soils. A combination of desalting and competitive binders increased yields, but again the increase was minimal, and we found that random hexamer primer loads greater than 5 μg began to inhibit DNA extraction.

Table 1 compiles the proposed soil-DNA interactions identified in this study.

### 3.5. Soil carryover

**FIG. 9.**
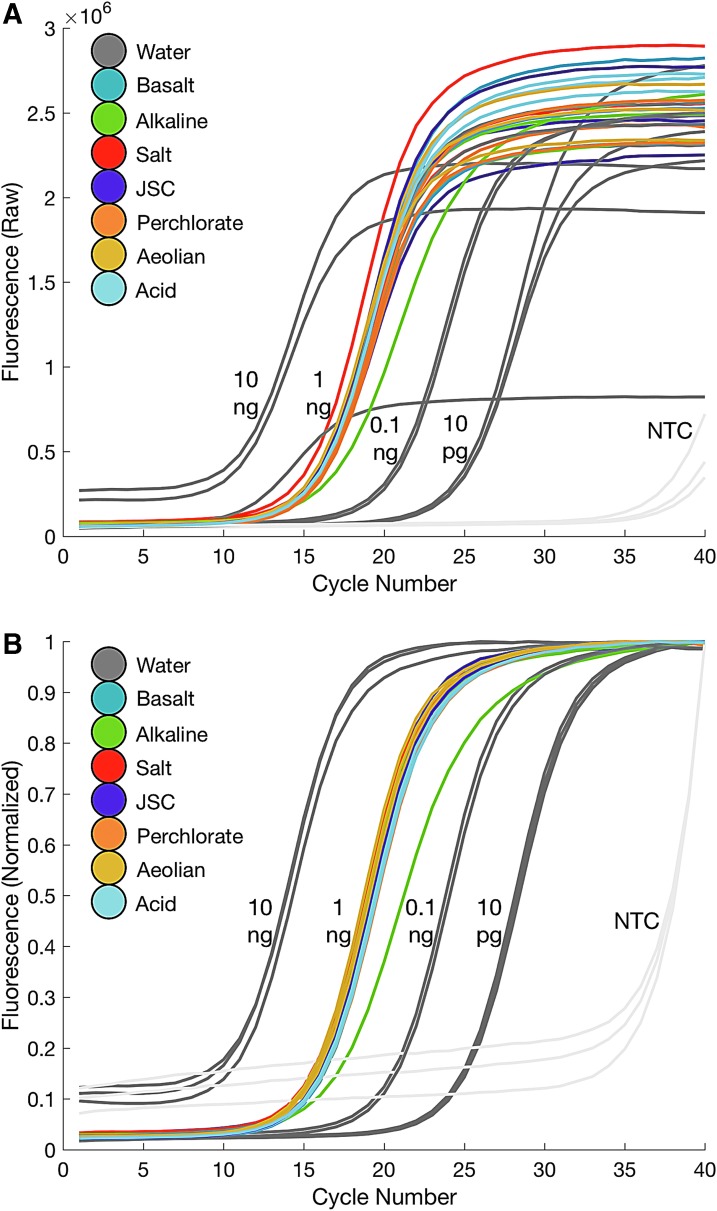
Quantitative PCR (qPCR) amplification curves reveal successful amplification from all synthetic Mars analog soils (perchlorate, alkaline, aeolian, acid, salt, JSC, and basalt) minus one alkaline sample (likely due to pipetting variability) suggest high PureLyse elution purity (1 ng of *B. subtilis* DNA, 10 ng to 10 pg and NTC standards, *n* = 3). (**A**) Raw fluorescence (**B**) Normalized fluorescence.

We observed successful qPCR amplifications from all simulated extractions containing 1 ng of *B. subtilis* DNA (added post-extraction) (Fig. 9), suggesting high DNA elution purity from PureLyse.

## 4. Discussion

### 4.1. Extraction and recovery of DNA from synthetic Mars analog soils

Here, we demonstrate that, with minimal soil-specific modifications, PureLyse Bacterial gDNA Extraction Kits are capable of extracting PCR-quality DNA from synthetic Mars analog soils representing a variety of martian environments (Figs. 8 and 9). We confirm that DNA extraction difficulties primarily derive from binding by free cations, adsorption to minerals, and destruction due to hydroxyl radicals and high acidity. Our results suggest that desalting soils prior to extraction is an effective approach for purging highly disruptive cations such as Fe^3+,2+^ (participate in redox cycling, bind DNA) and Ca^2+^ (bind DNA) along with additional inhibitory ions (*e.g.,* Cl^-^, Na^+^, ClO_4_^-^, CO_3_^+^, SO_4_^-^, HSO_4_^-^). In addition, coating mineral adsorption sites with competitive binders also increases DNA yields. Random hexamer primers and skim milk were both effective competitors; however, we recognize that skim milk exhibits a higher affinity toward soils, while random hexamer primers appear nonselective and may actually inhibit DNA extraction if hexamer quantities are greater than the combined soil-PureLyse carrying capacity. Many of our experiments here focus on addressing single soil-DNA interactions, such as the basalt (adsorption) and alkaline (Ca^2+^ binding) soils. Further optimization focused on soils containing multiple soil-DNA interactions like the aeolian, perchlorate, and acid soil may further increase yields. We conclude that a combination of desalting and employing competitive binders is the most effective method for extracting DNA, of the conditions tested, and may be a suitable starting point with which to approach the development of a universal martian soil DNA extraction protocol.

### 4.2. Environmental analogues and limits of detection

Our current experiments rely on appreciable amounts of DNA that establish resolvable soil-DNA interaction profiles (Figs. 6 and 7). However, we must note that the large spore populations used in this study (2.2 × 10^8^ spores per 50 mg soil, ∼1 μg DNA) are unlikely to be observed in any terrestrial Mars analog environment or on Mars itself. Studies of microbial communities in the Atacama Desert and Antarctic McMurdo Dry Valleys (MDV) have reported detectable microbial populations as low as 500–5000 spores per 50 mg of soil (1 × 10^3^ cfu per gram of soil), although about 10^4^ to 10^6^ cells per gram of soil is more typical (Navarro-Gonzalez *et al.,* 2003; Goordial *et al.,* 2016). Current environmental analog testing is focused on optimizing extraction protocols with Atacama/MDV-like microbial populations in the synthetic Mars analog soils and environmental samples from Mars-relevant sites. The OmniLyse cell-lysis component of the PureLyse Bacterial gDNA Extraction Kits has previously demonstrated the ability to lyse down to 50 spores of *B. subtilis* and has been characterized as the “best yield” extraction method by the United States Department of Agriculture in comparison to 11 other extraction methods (Irwin *et al.,* 2014). Prior validation of OmniLyse suggests that DNA extraction of low spore populations analogous to Mars-like environments is feasible.

### 4.3. Small acid-soluble proteins and Mars

Small acid-soluble proteins (SASPs) within spores of *B. subtilis* bind to DNA and furnish protection from heat, high salts, desiccation, and UV radiation (Moeller *et al.,* 2009, 2012). SASPs may contribute to the resilience of *B. subtilis* spores, and if life exists on Mars today, it is reasonable to consider that some martian organisms may have inherited or evolved homologous or analogous sporulation-protective programs that could also affect simple DNA isolation protocols. Thus, we must carefully review this possibility as we mature our Mars extraction protocols. To mitigate the effect of SASPs, we also developed an “on the fly” protein separation protocol involving a 5% phenol solution that increases DNA yields from upward of 14.7% to 43% (Fig. 8). It may be possible to further mitigate protein interactions and increase DNA yields from *B. subtilis* with a phenol-compatible DNA extraction kit, although phenol may not be acceptable aboard a Mars payload due to its potential risk for organic contamination to other instruments. Phenol modifications were purposely excluded from our spore DNA extraction from soils due to human health concerns with large-n experiments. However, it may yet be possible to develop an alternative solution that uses the same principle to separate DNA and protein. In this case, significant increases in extraction yield may be possible for the synthetic Mars analog soils. However, the ability to form spores is not common in bacterial phylogeny; it is mainly found in Gram-positive Firmicute bacteria (Traag *et al.,* 2013).

### 4.4. DNA elution purity and sequencing

Recovering DNA does not always mean success (Barton *et al.,* 2006). Soil contaminants (*e.g.,* metal ions) can coelute with DNA and inhibit downstream analysis (*e.g.,* PCR, sequencing) without preventative or additional purification steps. We determined that the PureLyse elution is of high purity (Fig. 9) and may not contain any compounds that inhibit certain nanopore sequencing technologies that rely on PCR-participating enzymes. While in this study we employed universal 16s rRNA primers, future studies, including those with low spore populations, can utilize single-copy *B. subtilis* specific primers in order to resolve discrete spore DNA extraction yields at low cell counts, even in the presence of exogenous contamination. In addition, Takada-Hoshino and Matsumoto (2004) and Barton *et al.* (2006) showed that samples treated with synthetic DNA (poly-dIdC) or skim milk, competitive binders used to increase DNA yields, do not inhibit amplification and thus may not inhibit nanopore sequencing either.

## 5. Conclusions

Life on Mars, if it exists or existed in the not-so-distant past, may potentially be detected via *in situ* nucleic acid extraction and sequencing aboard a future Mars rover/lander. Martian metagenomics could conceivably identify extraterrestrial genomes and discern conserved regions that may also suggest a Mars-Earth shared ancestry (Ruvkun *et al.,* 2002; Isenbarger *et al.,* 2008). Here, we have developed methodologies that enable such downstream nucleic acid detection or analysis technologies for future Mars exploration. The results indicate soil chemistry must be considered when extracting nucleic acids from Mars analogues or future Mars samples. Substantial challenges remain to be addressed, mainly engineering of an automated system and a Mars-compatible lysis and extraction module that incorporates all key steps for successful DNA extraction (desalting, competitive binding, DNA-protein separation), and utilizing biological reagents in the context of planetary protection regulations. Our work suggests that nucleic acid–based life detection on Mars may be feasible in the context of extant or recently dead life-intact cells; however, extracellular DNA may be bound to minerals or destroyed once hydrated. Additional work is required to understand the precise conditions under which DNA bound to minerals can be displaced to facilitate detection. Similar challenges apply to preservation and extraction of ancient DNA on Earth, with the added challenge of warm temperatures and their associated high hydrolysis rates.

## Abbreviations Used

EDTA: ethylenediaminetetraacetic acid
EGTA: ethylene glycol tetraacetic acid
MDV: McMurdo Dry Valleys
qPCR: quantitative real-time polymerase chain reaction
SASPs: small acid-soluble proteins
SETG: Search for Extra-Terrestrial Genomes
